# Effects of Monobutyl and Di(*n*-butyl) Phthalate *in Vitro* on Steroidogenesis and Leydig Cell Aggregation in Fetal Testis Explants from the Rat: Comparison with Effects *in Vivo* in the Fetal Rat and Neonatal Marmoset and *in Vitro* in the Human

**DOI:** 10.1289/ehp.9490

**Published:** 2006-12-19

**Authors:** Nina Hallmark, Marion Walker, Chris McKinnell, I. Kim Mahood, Hayley Scott, Rosemary Bayne, Shiona Coutts, Richard A. Anderson, Irene Greig, Keith Morris, Richard M. Sharpe

**Affiliations:** MRC Human Reproductive Sciences Unit, Centre for Reproductive Biology, Queen’s Medical Research Institute, Edinburgh, United Kingdom

**Keywords:** compensatory Leydig cell failure, di(*n*-butyl) phthalate, fetal testis, human, *in vitro*, Leydig cell, Leydig cell aggregation, marmoset, rat, testosterone

## Abstract

**Background:**

Certain phthalates can impair Leydig cell distribution and steroidogenesis in the fetal rat *in utero*, but it is unknown whether similar effects might occur in the human.

**Objectives:**

Our aim in this study was to investigate the effects of di(*n*-butyl) phthalate (DBP), or its metabolite monobutyl phthalate (MBP), on testosterone production and Leydig cell aggregation (LCA) in fetal testis explants from the rat and human, and to compare the results with *in vivo* findings for DBP-exposed rats. We also wanted to determine if DBP/MBP affects testosterone production *in vivo* in the neonatal male marmoset.

**Methods:**

Fetal testis explants obtained from the rat [gestation day (GD)19.5] and from the human (15–19 weeks of gestation) were cultured for 24–48 hr with or without human chorionic gonadotropin (hCG) or 22R-hydroxycholesterol (22R-OH), and with or without DBP/MBP. Pregnant rats and neonatal male marmosets were dosed with 500 mg/kg/day DBP or MBP.

**Results:**

Exposure of rats *in utero* to DBP (500 mg/kg/day) for 48 hr before GD21.5 induced major suppression of intratesticular testosterone levels and cytochrome P450 side chain cleavage enzyme (P450scc) expression; this short-term treatment induced LCA, but was less marked than longer term (GD13.5–20.5) DBP treatment. *In vitro*, MBP (10^−3^ M) did not affect basal or 22R-OH-stimulated testosterone production by fetal rat testis explants but slightly attenuated hCG-stimulated steroidogenesis; MBP induced minor LCA *in vitro*. None of these parameters were affected in human fetal testis explants cultured with 10^−3^ M MBP for up to 48 hr. Because the *in vivo* effects of DBP/MBP were not reproduced *in vitro* in the rat, the absence of MBP effects *in vitro* on fetal human testes is inconclusive. In newborn (Day 2–7) marmosets, administration of a single dose of 500 mg/kg MBP significantly (*p* = 0.019) suppressed blood testosterone levels 5 hr later. Similar treatment of newborn co-twin male marmosets for 14 days resulted in increased Leydig cell volume per testis (*p* = 0.011), compared with co-twin controls; this is consistent with MBP-induced inhibition of steroidogenesis followed by compensatory Leydig cell hyperplasia/hypertrophy.

**Conclusions:**

These findings suggest that MBP/DBP suppresses steroidogenesis by fetal-type Leydig cells in primates as in rodents, but this cannot be studied *in vitro*.

Disorders of male reproductive health, including testicular cancer, cryptorchidism, hypospadias, and low sperm counts, are common and may be increasing in incidence in the Western World (Sharpe and Skakkebaek 2003; Skakkebaek et al. 2001). Although testicular germ cell cancer and low sperm counts do not manifest until adulthood, there is increasing evidence that these disorders, as well as cryptorchidism and hypospadias, may have their origins in fetal life (Sharpe 2006; Skakkebaek et al. 2001). Based on such observations, the aforementioned disorders have been hypothesized to comprise a testicular dysgenesis syndrome (TDS), which stems from dysfunction of the Leydig and/or Sertoli cells in the fetal testis (Sharpe 2006; Sharpe and Skakkebaek 2003; Skakkebaek et al. 2001).

We and others have shown that fetal exposure of male rats to di(*n*-butyl) phthalate (DBP), or to certain other phthalates, induces TDS-like effects (Barlow and Foster 2003; Ema et al. 2000; Mylchreest et al. 2000), and this may provide an animal model in which the events in the human fetal testis that give rise to TDS can be investigated (Fisher et al. 2003; Mahood et al. 2005). Moreover, because human exposure to phthalates is substantial and widespread (Hauser and Calafat 2005; Koch et al. 2006; Silva et al. 2004), it is possible that such exposures might be a cause of TDS disorders in humans, although there is presently only one piece of evidence to support this possibility (Swan et al. 2005). One of the main effects of DBP and certain other phthalates on the fetal rat testis is the suppression of testosterone production via effects on cholesterol uptake, transport, and steroidogenic enzyme expression (Barlow et al. 2003; Parks et al. 2000). Therefore, one approach toward evaluating whether phthalates can adversely affect the fetal human testis would be to establish whether similar suppression of steroidogenesis, as occurs *in vivo* in the fetal rat testis, can be induced *in vitro* in rat and human fetal testis explants by DBP or its main active metabolite monobutyl phthalate (MBP). This was the primary initial aim of the present studies.

As well as inhibiting testosterone production and expression of insulin-like factor-3 (Insl3) (McKinnell et al. 2005; Wilson et al. 2004) by fetal Leydig cells in the rat, *in utero* exposure to DBP also induces abnormal fetal Leydig cell aggregation (LCA)/migration (Mahood et al. 2005). This in turn leads to focal areas of dysgenesis within otherwise normal testes, typified by malformed tubules and intratubular Leydig cells (Fisher et al. 2003; Mahood et al. 2005); in adulthood, such tubules lack germ cells (Mahood et al. 2005). As similar aberrant morphology has been reported in noncancerous regions of testes from men with testicular cancer (Hoei-Hansen et al. 2003; Sharpe 2006), which is considered to be the severest form of TDS, we have hypothesized that such “focal dysgenesis” may be one of the events that leads to or reflects altered Leydig (and perhaps Sertoli) cell function with consequent downstream effects, namely TDS disorders (Mahood et al. 2005, 2006). In this respect, focal (abnormal) LCA in the fetal testis can be viewed as an indicator of dysgenesis and of potential TDS disorders, so the demonstration that DBP/MBP could induce LCA *in vitro* in cultured fetal testes would provide an indirect way of assessing the ability of these chemicals to induce TDS.

The aim of the present studies was therefore 2-fold. First, we wanted to determine whether DBP, or its main metabolite MBP, could induce Leydig cell effects *in vitro* in the rat comparable to those observed *in vivo*; such a demonstration would enable easier study of the mechanisms via which such effects occur. As a prelude to undertaking such studies, we considered it essential to establish if DBP treatment *in vivo* within the same 48-hr time frame as used for the optimized fetal rat testis cultures [gestation day (GD)19.5–21.5] could inhibit steroidogenesis and/or induce fetal LCA. Second, we wanted to establish whether similar effects of MBP/DBP were demonstrable *in vitro* using human fetal testis explants. Finally, because only limited studies with human fetal testes *in vitro* are possible, we also evaluated whether MBP treatment of the neonatal marmoset was able to affect Leydig cell function during the neonatal testosterone rise.

## Materials and Methods

### Animals, treatments, sample collection, and processing

#### Rats

Wistar rats were maintained in our own animal facility and were fed *ad libitum* with a soy-free breeding diet (SDS, Dundee, Scotland). Adult females that had been time-mated were treated from GD13.5–20.5 with either corn oil vehicle (control) or 500 mg/kg DBP (99% pure; Sigma-Aldrich Ltd., Dorset, UK) in 1 mL/kg corn oil administered daily by oral gavage; this treatment regimen corresponds to the schedule we used previously (Fisher et al. 2003; Mahood et al. 2005) and is termed “long-term DBP treatment.” To test whether similar effects to those induced by this treatment could be induced by short-term DBP treatment, pregnant females were treated only on GD19.5–20.5. In both instances, animals were then killed by inhalation of carbon dioxide on GD21.5, and fetuses were removed, decapitated, and placed in ice-cold phosphate-buffered saline (PBS; Sigma-Aldrich). There was a minimum of three treated females in each group. Testes were removed via microdissection and fixed for 1 hr in Bouins fixative before being transferred to 70% ethanol. Testes were weighed and then processed into paraffin wax using standard methods and an automatic Leica processor (Leica Microsystems, Milton Keynes, UK).

#### Marmosets

Animals were captive-bred common marmoset monkeys (*Callithrix jacchus*), maintained in a colony that has been self-sustaining since 1973. Two studies were undertaken. In the first, 10 newborn marmosets were used, comprising five pairs of co-twin males. Marmosets show considerable between-animal variability that would normally necessitate the use of large numbers of animals based on power calculations, but the use of a co-twin study design (one twin treated with vehicle, one twin treated with MBP) enabled pair-wise evaluation using smaller numbers of animals (Sharpe et al. 2002). Commencing at 4 days of age, infant marmosets were administered vehicle or 500 mg/kg/day MBP (P1132; TCI Europe, Zwijndrecht, Belgium). This time frame is a critical window for testis development (Grumbach 2005; Mann and Fraser 1996). MBP is considered the main and active metabolite of DBP within the body; MBP, rather than DBP, was administered to marmosets in the main studies because they have been reported to be relatively poor metabolizers of orally administered phthalate diesters (Rhodes et al. 1983, 1986). In the context of the present studies, the aim was to establish whether DBP/MBP could induce effects on Leydig cells in the marmoset comparable to those in the rat. For treatment, the MBP powder was dissolved in dimethyl sulfoxide, then suspended in honey. The MBP solution was then taken up into a 1-mL syringe fitted with soft silastic tubing at the end, and the appropriate dose was offered to the marmoset, which then took it by sipping from the end of the silastic tubing. Control co-twins were similarly treated with vehicle only. There were no major problems with this mode of treatment, which we have used previously for studies involving feeding with soy formula (Sharpe et al. 2002). For comparison, four non–co-twin marmosets 4–6 days of age were similarly treated for 14 days with 500 mg/kg/day DBP administered by the same method as MBP, and data obtained was compared with the control males from the co-twin study. Animals were killed 4 hr after the last treatment via the intraperitoneal injection of an overdose of sodium pentobarbitone (Euthatal; Rhone Merieux Ltd., Harlow, Essex, UK). At death, a heparinized blood sample was obtained by cardiac puncture; the plasma was then separated by centrifugation and stored at −20°C until used for the testosterone assay. The testes were then dissected out, fixed for 6 hr in Bouins before processing as described above for rat testicular samples. Testes were weighed after fixation in Bouins and before their transfer into 70% ethanol.

In the second study, we assessed the acute effects of MBP. Nine males 2–7 days of age were administered a single oral dose of 500 mg/kg MBP as described above, and a blood sample was collected 5 hr later for measurement of testosterone levels. Controls (*n* = 6) received vehicle alone.

For the studies above, animals were treated humanely and with regard for alleviation of suffering. All studies were performed according to the Animal (Scientific Procedures) Act (1986) under Project Licence approval by the UK Home Office. The studies in marmosets were also approved by the local ethical committee for studies in primates.

### Rat fetal testis explants

Fetal testis cultures used tissue obtained from GD19.5 fetuses from untreated, time-mated females. Recovered testes were microdissected using a binocular dissecting microscope (Leica, MZ6) with a transilluminated stage and external cold lights. Each testis was microdissected into 5–7 pieces by cutting along a transverse plane such that each testis included portions of the coelomic epithelium and the region adjacent to the mesonephros. Explants were cultured in the wells of a 24-well culture plate and were placed on a porous membrane (0.45 μm) raised on a plastic scaffold within the culture well. Pre-warmed (37°C) medium was added, 0.2 mL to the well and 0.2 mL within the scaffold, to immerse the explants nesting on the insert membrane. Explants were distributed evenly between the wells per experiment with a maximum of five per insert. Replicates of both control and treated explants were run side by side under identical conditions. Cultures were incubated at 37°C for 48 hr in a humidified atmosphere of 95% air and 5% CO_2_.

In a range of preliminary studies, culture conditions were optimized based on the following end points: *a*) preservation of normal tissue architecture without major apoptosis or necrosis; *b*) maintenance of a high proliferation index (~ 30%) for Sertoli cells; and *c*) maintenance of steroidogenic responsiveness to added stimuli. These end points were evaluated by *a*) fixation of tissue explants in Bouins and their analysis after immunostaining for cell-specific markers as described previously (Mahood et al. 2005, 2006; McKinnell et al. 2005); *b*) by measurement of the incorporation of bromodeoxyuridine (BrdU), which was added to the incubation media for the last 4 hr of culture, and *c*) by measurement of testosterone secreted into the culture media over the 48-hr culture period. Different culture media and additions, different culture periods, and different age of fetal testes were among the parameters investigated during the optimization experiments.

As a result of these studies, we used the following final protocol. We used Dulbecco’s minimal essential medium with F12 (Gibco 2104-025; Invitrogen, Paisley, UK) to which was added sodium pyruvate (S8636; Sigma), bovine serum albumin (BSA; A2153; Sigma), and an insulin–transferrin sodium–selenite media supplement (×100; I3146; Sigma). Explants from GD19.5 fetuses that were cultured for 48 hr were used throughout. Under the culture conditions specified, tissue architecture was largely maintained and a Sertoli cell proliferation index > 30% was demonstrable (data not shown). Steroidogenic responsiveness to both human chorionic gonadotropin (hCG) and 22R-hydroxycholesterol (22R-CHO) was also maintained. For addition to the medium, we used the following compounds and concentrations: hCG (Profasi; Serono, Rockland, MA, USA) at 0.1 IU/mL; 22R-CHO (H9384; Sigma) at 50 μM; ketoconazole (KTZ; K1003; Sigma) at 100 μM; and BrdU (B9285; Sigma) at 10 mg/mL. All of these compounds were diluted in culture medium before addition, with the exception of KTZ, which was pre-dissolved at 100× target concentration in 70% ethanol (vol/vol medium). Additionally, MBP or DBP were added to culture media at concentrations ranging from 10^−6^ M to 10^−3^ M in 0.027% dimethyl sulfoxide. Appropriate volumes of solvent used for the additions were added to other cultures that served as basal controls.

### Human fetal testis explants

Human testes were obtained after medical termination of pregnancy at 15–20 weeks of gestation. None of the terminations were for reasons of fetal abnormality, and all fetuses used for the present studies appeared morphologically normal. Fetal testes were microdissected and placed into culture within 2–4 hr of removal from the fetus and were maintained on ice prior to use. Ethical permission for these studies was granted by the Lothian Paediatrics/Reproductive Medicine ethics subcommittee, and informed consent was obtained from each of the female patients involved according to national guidelines (Polkinghorne 1989). The preparation and culture of the human fetal testis explants used procedures identical to those in the rat; because the testes were larger, many more explants were obtained per testis, and these could not be sectioned along any particular plane as for the rat. The stages of gestation for which human fetal testis tissue was available broadly corresponds to the stages used for the rat fetal testis studies *in vitro* in that it encompasses the period of maximal testosterone production by the fetal Leydig cells as well as masculinization of the fetus.

### Stereological analysis of marmoset testes

Sections of testes from vehicle- and MBP-exposed co-twins and DBP-exposed singleton males were immunostained for 3β-hydroxy-steroid dehydrogenase (3β-HSD) and then subjected to stereological analysis, as described previously (Sharpe et al. 2002; Tan et al. 2006), to determine the number of 3β-HSD–immunopositive Leydig cells and their cytoplasmic volume.

### Testosterone measurement

Levels of testosterone from blood and testes and in culture media from rat and human testis explants were measured by radioimmunoassay, as described previously (Mahood et al. 2005). After dissection, testes were snap frozen on dry ice and stored at −70°C until analysis. Testes were defrosted and homogenized individually in 0.5 mL PBS; a 100-μL aliquot was then extracted with 2 mL diethyl ether, shaken for 5 min, and placed in a bath of methanol cooled with dry ice. The non-aqueous portion of the extract was then decanted, dried overnight in a fume hood, and reconstituted in appropriate buffer for the radioimmunoassay. Culture media were not extracted before the assay but were diluted as required in assay buffer. All samples from a particular experiment were assayed together in a single run. The limit of detection of the assay was 12 pg/mL.

### Statistical analysis

Data were analyzed using analysis of variance followed by the Bonferroni post-test, using GraphPad Prism (version 4; GraphPad Software Inc., San Diego, CA, USA). Marmoset co-twin data was analyzed using a paired *t*-test. Data for Leydig cell cluster analysis and relative testosterone production by testis explants were log transformed before analysis to normalize variances.

## Results

### *Comparison of short- and long-term exposure to DBP* in utero *in the rat.*

**Figure 1:**
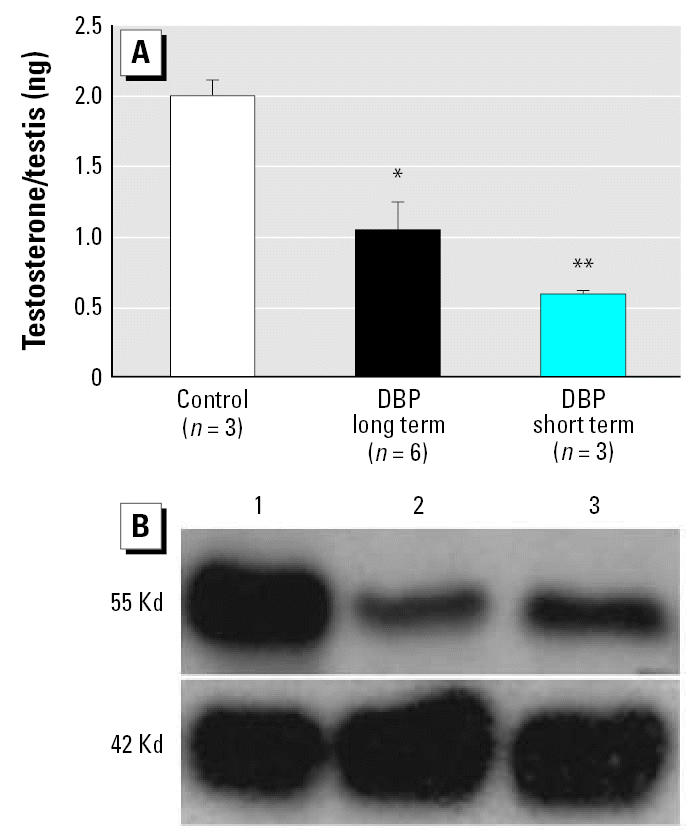
Comparison of short-term (GD19.5–20.5) and long-term (GD13.5–20.5) treatment with DBP on (*A*) testicular levels of testosterone and (*B*) the relative expression of P450scc (55 Kd band) compared with smooth muscle actin (42 Kd band), as assessed by Western blot at GD21.5. Values shown are mean ± SE for 3–6 testes per group. *p < 0.05, and **p < 0.01, compared with the respective control.

**Figure 2:**
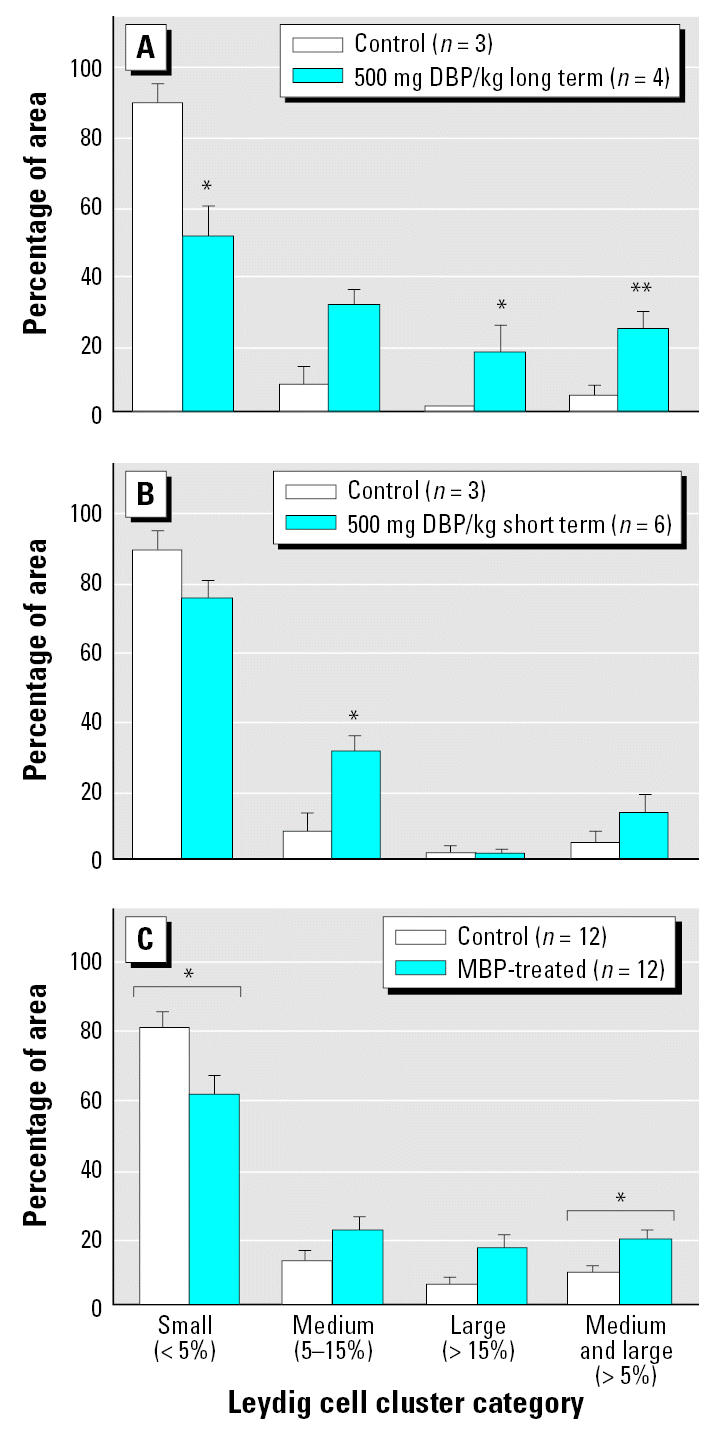
Effect of long-term (GD13.5–20.5; *A*) or short-term (GD19.5–20.5; *B*) treatment with DBP or *in vitro* exposure of GD19.5 fetal rat testis explants to 10^−3^ M MBP (*C*) on LCA; effects were determined on GD21.5 (*A*, *B*) or after 48 hr culture (*C*). Leydig cell cluster size was defined as follows: small clusters were those that accounted for ≤ 5% of the total Leydig cell cluster area per testis, medium clusters accounted for 5.1–14.9%, and large clusters individually accounted for ≥ 15%. Values shown are mean ± SE. *p < 0.05, and **p < 0.01, compared with the respective controls.

We used two primary end points to compare the effectiveness of short-term (GD19.5–21.5) and long-term (GD13.5–21.5) *in utero* DBP exposure, namely, testicular testosterone levels and the induction of fetal LCA—both of which have been shown previously to be key effects of DBP on the fetal rat testis *in vivo* (Barlow et al. 2003; Mahood et al. 2005; Parks et al. 2000). Our comparison showed that testicular testosterone levels and testicular expression of P450 side chain cleavage enzyme (P450scc) were reduced substantially by both short- and long-term treatment with DBP, with the shorter term treatment regimen inducing at least as large a reduction as the long-term treatment (Figure 1). Similarly, both treatment regimes induced significant LCA as judged by the shift in proportion of small Leydig cell clusters to medium or large Leydig cell clusters (Figure 2). However, the shorter-term DBP treatment regimen was noticeably less effective in inducing LCA compared with the longer-term DBP treatment regimen (Figure 2). On the basis of these findings, we concluded that primary Leydig cell effects of DBP treatment are inducible *in vivo* within the time window GD19.5–21.5 and that the use of explants from GD19.5 fetuses cultured for 48 hr should allow detection of similar effects *in vitro*, especially on steroidogenesis.

We also demonstrated that treatment of pregnant rats with 500 mg/kg/day MBP from GD13.5 to GD20.5 resulted in similar changes to the fetal testes of male offspring at GD21.5 (induction of LCA, induction of multinucleated gonocytes) as did treatment with DBP (data not shown).

### *Effects of MBP or DBP on testosterone production* in vitro *by rat fetal testis explants.*

**Figure 3:**
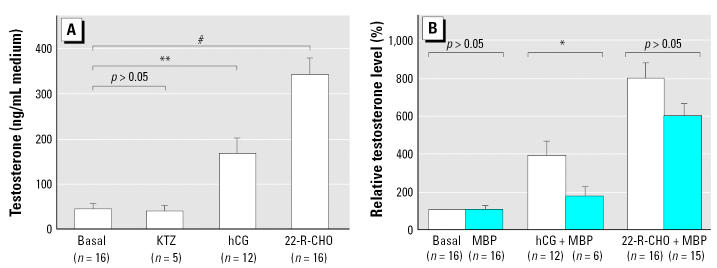
Testosterone production *in vitro* by GD19.5 rat testis explants after culture for 48 hr at 37°C with or without the addition of hCG, 22R-CHO, or KTZ in the presence or absence of 10^−3^ M MBP. Data in (*A*) is shown in terms of absolute testosterone production, whereas data in (*B*) is expressed as fold change from the respective basal sample. Values are mean ± SE for 3–17 cultures from separate fetuses per group. *p < 0.05, **p < 0.01, and ^#^*p* < 0.001, compared with basal control.

We found considerable variation in testosterone production by individual explant cultures, irrespective of the culture additions, and this necessitated the use of a reasonably high number of repeated experiments, which were then pooled for final analysis. In these cultures, the addition of hCG always increased testosterone production, with an average increase above basal levels of approximately 4-fold (Figure 3). Similarly, addition of 22R-CHO also increased testosterone production in a consistent manner, with levels being increased on average > 8-fold above basal levels (Figure 3). In contrast, addition of KTZ, which was added to demonstrate suppressibility of testosterone production, failed to consistently induce such a decrease over a 48-hr culture period compared with basal levels of testosterone production (Figure 3).

Addition of DBP at concentrations ≤ 10^−3^ M had no effect on basal or stimulated testosterone production (data not shown). Addition of MBP to the culture medium at concentrations of 10^−6^ M to 10^−4^ M (data not shown) or 10^−3^ M, had no consistent or significant effect on basal testosterone production (Figure 3). Similarly, addition of MBP at any of these concentrations failed to attenuate the increased testosterone production induced by addition of 22R-CHO (Figure 3). However, addition of 10^−3^ M MBP did slightly, but significantly (*p* < 0.05), attenuate hCG-stimulated testosterone production (Figure 3), although there was no significant effect observed when lower concentrations of MBP were added to cultures stimulated with hCG (data not shown).

### Effect of MBP on LCA in rat fetal testis explants

Addition of 10^−3^ M MBP slightly, but significantly (*p* < 0.05), caused a shift in Leydig cell cluster size away from small to medium/large (Figure 2).

### Effects of DBP or MBP on testosterone production by human fetal testis explants

**Figure 4:**
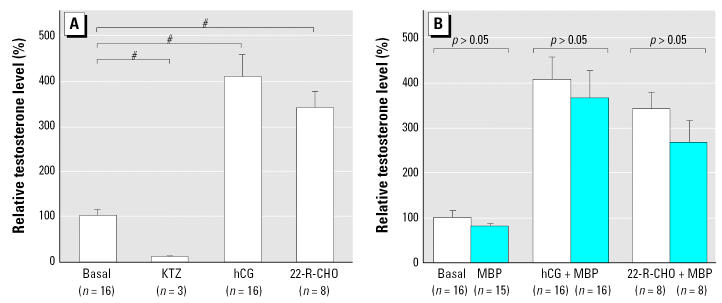
Testosterone production *in vitro* by fetal human testis explants after culture for 24 hr at 37°C with or without the addition of hCG, 22R-CHO, or KTZ in the presence or absence of 10^−3^ M MBP. Values are expressed as fold change from the respective basal sample and are the mean ± SE for 3–16 replicates from testes from four separate fetuses. ^#^*p* < 0.001, compared with basal value.

Using culture conditions identical to those used for the rat studies above, explants of fetal human testis were cultured in the presence or absence of 10^−3^ M MBP or DBP with or without the addition of hCG, 22R-CHO, or KTZ for up to 48 hr. Testosterone production by the human fetal testis explants over 48 hr of culture was highly variable, irrespective of gestational age or the culture additions. Addition of 10^−3^ M MBP or DBP under basal, hCG-, or 22R-CHO-stimulated conditions had no significant attenuating effect on testosterone production, although KTZ consistently and significantly reduced testosterone production by approximately 80% (data not shown). However, because histologic examination of most of these cultured explants showed evidence of central necrosis, we decided that shorter culture periods (1, 5, and 24 hr) should be evaluated to establish whether any effects were demonstrable under these conditions; results are shown for the 24 hr cultures (Figure 4). In these samples, hCG and 22R-CHO both significantly enhanced testosterone secretion above basal levels, and KTZ significantly reduced testosterone production; however, addition of 10^−3^ M MBP had no effect on testosterone secretion in the presence of hCG or 22R-CHO or under basal conditions (Figure 4).

### LCA in human fetal testis explants

Because of the dispersed distribution of Leydig cells in human tissue compared with rat tissue, it was not possible to perform computer-assisted image analysis of Leydig cell clusters in the human fetal testis explants as for the rat; therefore, no conclusions could be drawn as to whether MBP could induce LCA *in vitro* in the human. However, visual inspection of sections of cultured testes immunostained for 3β-HSD revealed no evidence of conspicuous LCA (not shown).

### *Effect of MBP or DBP* in vivo *on testosterone levels and Leydig cell volume in the neonatal marmoset.*

**Figure 5:**
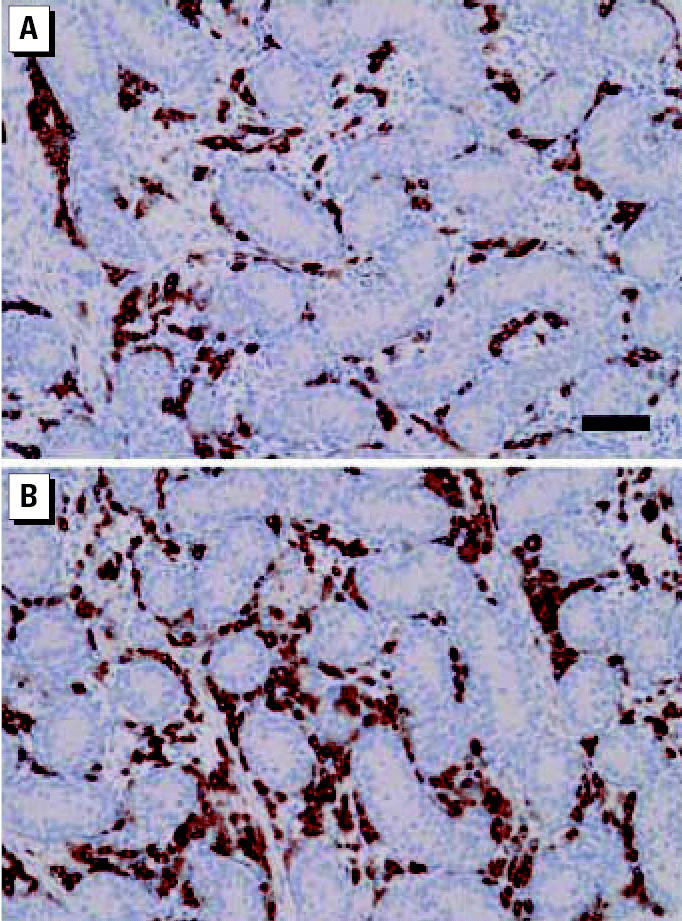
Representative testicular histology and occurrence of Leydig (3β-HSD–immunopositive) cells in the testes of neonatal marmoset co-twins (co-twins number 5 in Figure 6) after treatment for 14 days with either vehicle (control twin, *A*) or 500 mg/kg/day MBP (*B*). Note the apparently greater number or size of the Leydig cells in the MBP-exposed animal. Bar = 100 μm and applies to both images.

**Figure 6:**
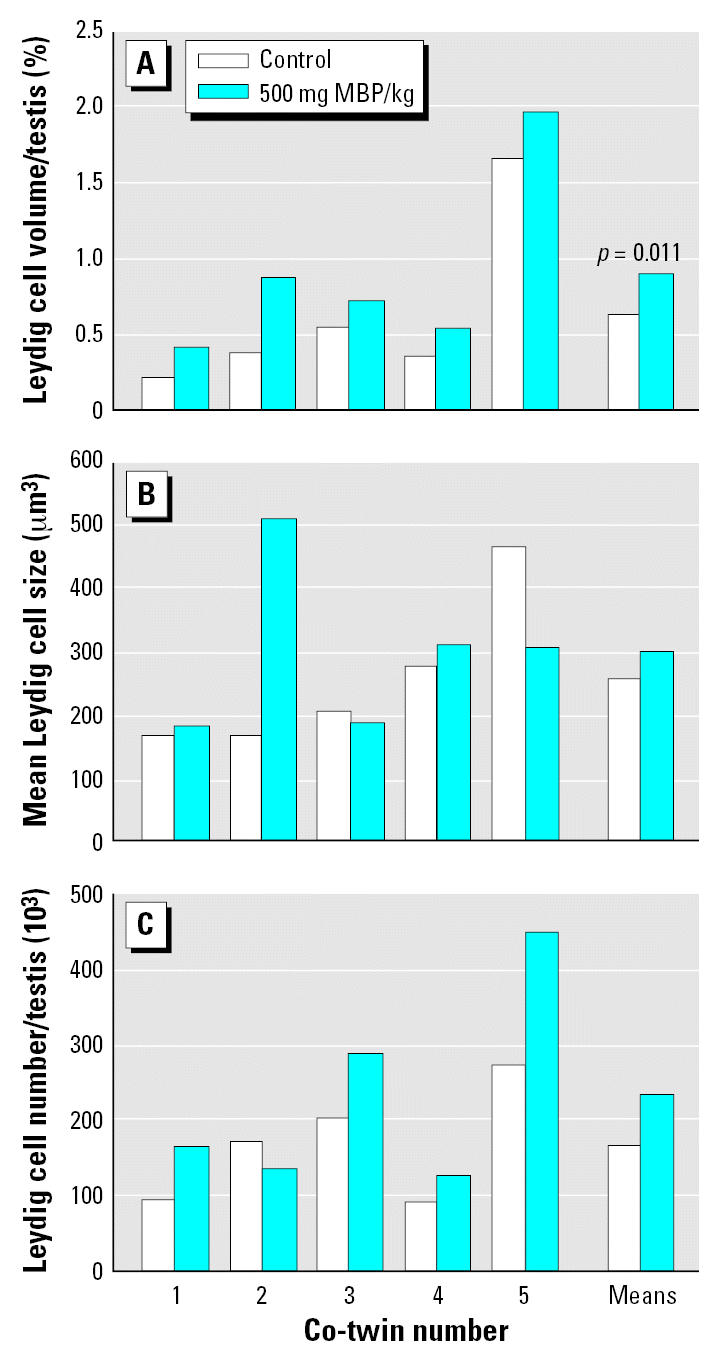
Leydig cell parameters in neonatal marmoset co-twins treated for 14 days with either vehicle (control twin) or 500 mg MBP/kg/day. (*A*) Leydig cell volume/testis. (*B*) Average Leydig cell size. (*C*) Total Leydig cell number per testis. The paired *t*-test was used for statistical comparison.

Co-twin marmosets were treated with either vehicle or 500 mg/kg/day MBP for 14 days, commencing 4 days after birth. Plasma levels of testosterone measured at the end of the study period revealed no significant change between control and MBP-treated co-twins (controls, 3.3 ± 1.4 ng/mL; MBP, 3.3 ± 2.1 ng/mL; mean ± SE; *n* = 5). Testis weight was slightly reduced in MBP-treated co-twins (10.8 ± 1.2 mg) compared with their co-twin controls (11.5 ± 1.0 mg), but this difference was inconsistent and nonsignificant. However, it appeared from sections immunostained for 3β-HSD that Leydig cell volume per testis was increased (Figure 5). This was confirmed by stereological analysis, which demonstrated that the total volume of Leydig cells (cytoplasm plus nucleus) per testis was consistently and significantly (*p* = 0.011) increased in MBP-treated animals compared with their co-twin controls; this change was due to an increase in cell number in four of the MBP-treated males and to a trebling in Leydig cell cytoplasmic volume in the fifth MBP-treated animal (co-twin number 2) when compared with its co-twin control (Figure 6). Similarly, in four marmosets treated with DBP rather than MBP, a similar increase in Leydig cell volume per testis (1.26 ± 0.17%) was evident when compared with values for control marmosets (0.65 ± 0.26%) from the MBP co-twin study, although this difference did not reach statistical significance (Student *t*-test). The increase in Leydig cell volume induced by MBP/DBP was probably attributable to increased stimulation by raised luteinizng hormone (LH) levels resulting from initial suppression of steroidogenesis by the MBP treatment, but this could not be confirmed directly because of the lack of availability of an assay for marmoset LH. Therefore, nine male marmosets 2–7 days of age were administered a single oral dose of 500 mg/kg MBP or vehicle (controls, *n* = 6) and blood was collected 5 hr later. This showed that testosterone levels were significantly (*p* = 0.019) suppressed in MBP-treated animals (1.36 ± 0.23 ng/mL; mean ± SE) compared with controls (2.75 ± 0.55 ng/mL).

## Discussion

The primary objectives of the present studies were *a*) to establish if Leydig cell effects of DBP/MBP could be demonstrated *in vitro* using rat fetal testis explants, and *b*) once such a culture method had been established and validated, to undertake comparable studies using human fetal testis explants to establish if DBP/MBP could exert effects in the human comparable with those described in the rat. However, although our studies using fetal testis explants from GD19.5 rats showed that addition of 10^−3^ M MBP was able to significantly attenuate hCG-stimulated testosterone production, this was a relatively minor effect and there was no effect of MBP on basal or 22R-CHO–stimulated testosterone production by rat fetal testis explants. Similarly, MBP induced only minor evidence of LCA *in vitro*, whereas *in vivo* DBP treatment beginning on GD19.5 was able to induce significant LCA by GD21.5. There was no attenuating effect of MBP on testosterone production by fetal human testis explants. Whether this reflects a genuine lack of effect or is indicative of problems with the testis explant approach is discussed further below. In view of such uncertainties, we explored an alternative approach to evaluating whether DBP/MBP can affect steroidogenesis by fetal Leydig cells in primates by undertaking studies in neonatal male marmosets, the first involving MBP treatment of co-twins for a 2-week period during the neonatal testosterone rise. Although this study failed to demonstrate any significant suppression of blood testosterone levels after 2 weeks of treatment, it did provide striking, indirect evidence that MBP exerted negative effects on the fetal Leydig cells. This was confirmed by a follow-up study in which a single treatment with 500 mg/kg MBP significantly suppressed blood testosterone levels in neonatal marmosets.

Addition of 10^−3^ M MBP significantly attenuated hCG-stimulated, but not 22R-CHO–stimulated, testosterone production by rat fetal testis explants. This contrast is consistent with the known mechanisms through which DBP/MBP inhibits steroidogenesis by fetal rat Leydig cells *in vivo*. Thus, DBP exposure *in utero* reduces expression of genes encoding proteins involved in cholesterol uptake and transport into the mitochondrion, as well as suppressing P450scc and P45017-hydroxylase (Barlow et al. 2003; Lehmann et al. 2004). Because this mode of action affects pathways immediately downstream of the LH receptor, it would perhaps be expected that MBP would be able to attenuate hCG-stimulated testosterone production. In contrast, because 22R-CHO bypasses the steps in steroidogenesis that involve transport of cholesterol into the mitochondrion, MBP may be unable to exert any major inhibitory effect on steroidogenesis distal to this step. Nevertheless, it is puzzling why MBP caused only minor attenuation of the hCG-induced increase in testosterone production and why it was unable to suppress basal testosterone production under the same circumstances, in view of the known pathways of its effects on steroidogenesis. This suggests that the explant culture system used in the present studies is unable to faithfully reproduce the main effects of DBP/MBP on steroidogenesis that are evident *in vivo*. In this regard, our findings demonstrate that short-term *in vivo* treatment with DBP on GD19.5–21.5, which matches the *in vitro* culture period we used, was able to cause major suppression of steroidogenesis *in vivo* in the rat. The latter finding is consistent with earlier studies that showed rapid effects of DBP on steroidogenesis *in vivo* by the fetal rat testis (Thompson et al. 2004, 2005).

The fetal human testis explant system used in the present studies initially used conditions identical to those used for the rat explant cultures, but was then modified to include earlier time points. Relatively modest stimulatory effects of hCG and 22R-CHO on testosterone production were demonstrable, whereas KTZ caused marked suppression of testosterone production, in contrast to its ineffectiveness in the fetal rat testis cultures. Whether these differences reflect genuine species differences in control of fetal testicular testosterone production or whether it reflects differences in the viability/functionality of the explant cultures is unclear. Nevertheless, under all culture conditions tested for fetal human testis explants, no significant attenuation by MBP of testosterone production was demonstrable. In view of these findings and the modest inhibitory effects of MBP on testosterone production by rat testis explants, we concluded that it is not possible to reproduce the *in vivo* effects of DBP/MBP on steroidogenesis using fetal testis explants *in vitro*. It is a matter for speculation whether this is a consequence of inadequacies of the *in vitro* approach or whether it indicates that metabolites of DBP other than MBP are the cause of reduced testosterone production *in vivo*. We therefore concluded that only *in vivo* studies with DBP/MBP could be viewed as being definitive and, as a consequence, we turned to the marmoset to investigate whether DBP/MBP could suppress steroidogenesis by fetal-like Leydig cells in a primate.

We chose newborn male marmosets because they exhibit a neonatal testosterone rise comparable to human males (Grumbach 2005; Mann and Fraser 1996; McKinnell et al. 2001) and because the Leydig cells that make this testosterone are thought to be fetal-type Leydig cells rather than adult-type Leydig cells (Huhtaniemi and Toppari 1995). Administration of MBP to co-twin marmosets neonatally for 2 weeks did not suppress blood levels of testosterone, but there were consistent changes in Leydig cell volume per testis due to increased Leydig cell number and/or size (increased cytoplasmic volume); similar changes were observed in marmosets treated with DBP. These findings suggest a compensatory change caused by increased LH stimulation of the Leydig cells (de Kretser 2004; Ochsenkuhn and de Kretser 2003), a change that probably results from initial suppression of steroidogenesis with consequent reduced negative feedback to the hypothalamus–pituitary unit. Unfortunately, there is no blood LH assay for the marmoset, so we were unable to confirm that LH levels were elevated. However, as an alternative, we showed that a single treatment of neonatal marmosets with 500 mg/kg MBP significantly lowered testosterone levels 5 hr later, as has been shown previously for DBP in the rat (Thompson et al. 2004).

The finding from the co-twin study in marmosets and comparison of the effects of DBP/MBP on steroidogenesis by rat and human fetal testis explants raises the important issue of species and age differences in the regulation of fetal/neonatal steroidogenesis. In the fetal rat testis, testosterone production is LH independent until at least GD19.5 of fetal life (El-Gehani et al. 1998; Habert et al. 2001; Habert and Picon 1982). Even more importantly, the negative feedback mechanisms that would trigger increased LH secretion in response to reduced testosterone production by the fetal testis are not operative until around this age (Habert and Picon 1982; Huhtaniemi and Toppari 1995). One consequence of this arrangement is that suppression of steroidogenesis (by DBP/MBP) will not lead to any compensatory change (i.e., an increase in LH secretion), at least not until the end of gestation.

For most of fetal life the human testis is exposed to plentiful amounts of hCG that might potentially override or compensate for inhibitory effects of MBP on steroidogenesis. After birth, it is well established that testosterone production in the neonatal rat, human, and marmoset is completely LH dependent (Grumbach 2005; Habert and Picon 1982; Mann and Fraser 1996). As a consequence, DBP/MBP-induced suppression of steroidogenesis in the neonatal marmoset will lead to reduced negative feedback and hence a compensatory increase in LH secretion to restore steroid production to normal levels. This state of “compensated Leydig cell failure” (de Kretser 2004) will obscure any primary adverse effect of phthalate exposure on Leydig cell steroidogenesis. Although we could not demonstrate this directly in the present studies using neonatal marmosets, our observations are consistent with such a cascade of effects, as elevated LH levels would be expected to increase Leydig cell cytoplasmic volume and or cell number per testis (Ewing and Zirkin 1983). Thus, initial inhibition of steroidogenesis by DBP/MBP may be compensated for so that the original effect (i.e., lowered testosterone production) is no longer evident. This should be taken into consideration when evaluating whether or not phthalates may induce adverse effects on fetal steroidogenesis in the human compared with those that are well described in rodents.

One of the main conclusions of the present studies is that use of fetal testis explants to study the mechanisms of action of DBP/MBP appears limited and is perhaps nonviable. Though we carefully optimized explant cultures for the fetal rat testis, their limited responsiveness to MBP, when contrasted with that observed *in vivo* within exactly the same time frame, raises doubts about the utility of the *in vitro* approach. Results with human fetal testis explants raised even greater concerns, and our finding that MBP has no inhibitory effect on fetal Leydig cell testosterone secretion in the human under such circumstances is not one that we consider to be robust. Indeed our studies with neonatal marmosets suggest that MBP/DBP does have adverse effects *in vivo* on Leydig cell steroidogenesis in neonatal primates, although the initial inhibitory effect of DBP/MBP on steroidogenesis may be subsequently compensated for; the ability to “compensate” may vary between species and between ages (fetal versus neonatal and/or postnatal). As *in vitro* approaches for the study of DBP/MBP effects on the fetal testis appear of little value, future studies *in vivo* in the fetal marmoset may offer the best direct approach to evaluating effects relevant to the human.
